# Electrical transportation mechanisms of molybdenum disulfide flakes-graphene quantum dots heterostructure embedded in polyvinylidene fluoride polymer

**DOI:** 10.1038/s41598-019-43279-3

**Published:** 2019-05-01

**Authors:** Poh Choon Ooi, Muhammad Aniq Shazni Mohammad Haniff, M. F. Mohd Razip Wee, Boon Tong Goh, Chang Fu Dee, Mohd Ambri Mohamed, Burhanuddin Yeop Majlis

**Affiliations:** 1 Institute of Microengineering and Nanoelectronic, Universiti Kebangsaan Malaysia, 43600 Bangi, Malaysia; 2 Advanced Devices Lab, MIMOS Berhad, Technology Park Malaysia, 57000 Kuala, Lumpur Malaysia; 3 Low Dimensional Materials Research Centre (LDMRC), Department of Physics, Faculty of Science, University of Malaya, 50603 Kuala, Lumpur Malaysia

**Keywords:** Electronic devices, Electronic devices

## Abstract

In the interest of the trend towards miniaturization of electronic gadgets, this study demonstrates a high-density data storage device with a very simple three-stacking layer consisting of only one charge trapping layer. A simple solution-processed technique has been used to fabricate the tristable non-volatile memory. The three-stacking layer was constructed in between two metals to form a two-terminal metal-insulator-metal structure. The fabricated device showed a large multilevel memory hysteresis window with a measured ON/OFF current ratio of 10^7^ that might be attributed to the high charge trapped in molybdenum disulphide (MoS_2_) flakes-graphene quantum dots (GQDs) heterostructure. Transmission electron microscopy was performed to examine the orientation of MoS_2_-GQD and mixture dispersion preparation method. The obtained electrical data was used further to speculate the possible transport mechanisms through the fabricated device by a curve fitting technique. Also, endurance cycle and retention tests were performed at room temperature to investigate the stability of the device.

## Introduction

Recently, lots of effort has been conducted to realize the possibility of polymer non-volatile memory (NVM) device to attain high density data storage with high retention time in order to fulfill the increasing demand of modern electronic products and to overcome the technical and physical limitation of conventional Si-based flash memory scaling^1,2^. It is common that for the researchers to design the NVM devices in metal-insulator-metal (MIM) structure with minimal down scaling to achieve high density data storage in a single device^3,4^. In general, to achieve multilevel switching states for higher data storage capability, the MIM structure, which consists of one type of nanoparticles (NPs) as charge trapping medium, is usually embedded in minimum two layers of polymer^4,5^. The switching mechanisms using nanoparticles embedded in polymer could be induced by an electric field, regularly of a diffusion of an electrochemically active metal from an electrode and migration of oxygen vacancies in an insulator towards the cathode^6–8^.

In this study, the possibility of obtaining higher data storage potential by tristable switching approach using the nanocomposite mixture of molybdenum disulfide (MoS_2_) flakes and graphene quantum dots (GQDs) embedded in polyvinylidene fluoride (PVDF) polymer will be demonstrated because relatively few studies have examined the information storage capability of these two 2D NPs in a MIM device structure, to the best of our knowledge. The device is designed deliberately with a three-stacking layer consisting of one charge trapping layer instead of at least two to attain high density charge storage. The MoS_2_-GQD heterostructure will be used as single charge trapping layer embedded in two insulator layers to create charge retention effect^9^. This could be the right step to reduce the number of stacking layers and consequently reduce the manufacturing contamination, less induced heat and attained device size miniaturization as compared to the reported work by Ooi *et al*.^4^, that comprises of two charge layers in their fabricated device. MoS_2_ and GQD, the 2D-NPs with dimensions down to atomic level, have attracted high attention due to their fascinating properties in wide range of developments on the next generation of miniaturized electronic applications such as optoelectronics, optically transparent, and mechanically flexible devices^10–14^.

MoS_2_ NPs have been identified as a promising material for switching device application due to its high work function, quantum confinement effect, a direct band gap of 1.8 eV in monolayer, and a high mobility of 200 cm^2^ V^−1^s^−1^ with a high current ON/OFF ratio at approximately 10^8^ when used as channel materials in field effect transistors geometry^15–17^. The monolayer MoS_2_ is very sensitive to the presence of charges but it demonstrates the drawbacks of relatively small hysteresis window with degraded mobility and insufficient charge trap capability that might be correlated to charge trap stacking issue when it is constructed in a MIM geometry^10,17^. Therefore, the nanometer-sized GQD will be integrated with MoS_2_ flake to create a MoS_2_-GQD heterostructure charge trap medium to enhance data storage capacity and retention time. This integration is possible because of semi-metallic properties of GQD that could form the ideal contact with MoS_2_, and thus capable of supporting large current density, exceeding 10^9^ A/m^2 ^^18–20^. Moreover, the lack of dangling bonds at the interface between graphene and those 2D semiconductors would suppress the formation of interface states and charge traps^18^.

As aforementioned, the blend of GQDs and MoS_2_ NPs will be dispersed in PVDF polymer to form a GQD-MoS_2_-PVDF nanocomposite (GMP-NC). PVDF polymer is used as dielectric barrier in this work owing to its flexibility, low weight, and low-cost solution processability. In addition, PVDF is also a non-reactive nature and possesses better heat resistance^21,22^. A switching device will be fabricated on indium-tin-oxide (ITO) coated glass substrate using simple solution process route in the structure of GMP-NC layer in between the PVDF dielectric layers. Solution-processing deposition is exploited as one of the promising methods to realize a low-cost and simple method for the construction of optically transparent and mechanically flexible electronic devices. Moreover, this method could avoid expensive and tedious fabrication process such as time-consuming operational steps, high-temperature mean, and costly vacuum environment^9,23^.

## Results

### Lattice-resolved characterization of GQDs-MoS_2_ heterostructure

**Figure 1:**
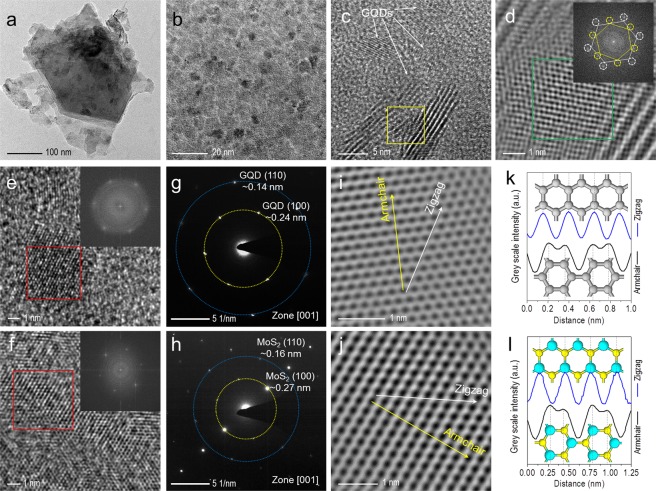
(**a**) TEM image of the MoS_2_ flake decorated with GQDs. (**b**) TEM image of the GQDs on the MoS_2_. (**c**) HR-TEM image of the GQDs on the few-layer MoS_2_. (**d**) Fourier filtered HR-TEM image of a single GQD/MoS_2_ heterostructure on the selected yellow square area in (**c**), inset of (**d**) is the fast Fourier transform (FFT) pattern displaying two sets of sixfold coordination symmetry corresponding to GQD (white) and MoS_2_ (yellow). (**e,f**) HR-TEM images of the GQDs and few-layer MoS_2_. Two insets in (**e**,**f**) showing their corresponding FFT patterns, respectively. (**g**,**h**) SAED images of the single-crystalline GQD and few-layer MoS_2_ observed along the [001] zone axis. (**i**,**j**) Fourier filtered HR-TEM image of the GQD and few-layered MoS_2_ on the selected red square area in (**e**,**f**). The dark spots indicating the void space. White and yellow lines representing the zigzag and armchair directions, respectively. (**k**,**l**) Profile plots of grey scale intensities for the GQD and few-layer MoS_2_ along the selected line as illustrated in (**I**,**j**). Grey, cyan and yellow atoms denoting the C, Mo, and S elements, respectively.

The atomic structure of the GQDs-MoS_2_ heterostructure that is incorporated into the fabricated device structure was characterized by high-resolution transmission electron microscopy (HR-TEM). Figure 1(a) displays a low-magnification bright-field TEM image of a MoS_2_ flake decorated with GQDs, illustrating a stacked few-layer MoS_2_ from the visibility of color contrast along the edges due to interlayer coupling behavior of the exfoliated natural crystals. Few folded and disorder regions can be observed at the edges, indicating possible lattice defects, dislocation, or local stresses in the MoS_2_ layer. A higher magnification image in Fig. 1(b) shows that the GQDs with different sizes up to ~10 nm is closely packed together on the MoS_2_ flake. In Fig. 1(c), high-resolution TEM shows multi-layer MoS_2_ with interlayer spacing of ~0.62 nm, which corresponds to the MoS_2_ (002) lattice plane. The GQDs are expected to be misaligned on the MoS_2_ flake, as evidenced by the Fourier filtered HR-TEM image in Fig. 1(d). Notably, the result shows clear Moiré patterns (indicated by the green square area) in between the MoS_2_ edges due to misorientation between a single GQD and MoS_2_ layers, which is further confirmed by fast Fourier transform (FFT) pattern of the HRTEM image, as shown by the insert in Fig. 1(d). At this region, two sets of hexagonally arranged diffraction spots that originated from GQD (white) and MoS_2_ (yellow) with a rotation angle of ~29.3° can be observed, indicate the single-crystalline nature of GQD and MoS_2_. Considering that MoS_2_ has larger lattice constant (a = 0.312 nm) than that of GQD (a = 0.246 nm), the diffraction spots from the first order of MoS_2_ should be located closer to the center of the electron beam. In addition, most of the GQDs are expected to be oriented randomly on the MoS_2_ flake by our dispersion method, which is further confirmed by typical Debye-Scherrer type ring pattern of graphene crystals from the FFT pattern (Refer to Supplementary Information, Fig. S1). HRTEM and selected area electron diffraction (SAED) characterization on individual GQD and MoS_2_ were also employed to further confirm the pristine nature of these 2D NPs. Both HRTEM images of GQD and few-layer MoS_2_ and their corresponding FFT patterns in Fig. 1(e,f) show the characteristic of single-crystalline lattice structure. Based on the corresponding SAED images with [001] zone axis, hexagonal lattice structures are clearly visible for both GQD and few-layer MoS_2_ as shown in Fig. 1(g,h), which are consistent with the literatures^24,25^. Here, the lattice spacing for the GQD can be evaluated to be ~0.25 nm and ~0.14 nm corresponding to the (100) and (110) planes, respectively. Meanwhile, the few-layer MoS_2_ reveals lattice spacing of ~0.27 nm and ~0.16 nm for the (100) and (110) planes, respectively. Furthermore, individual C, Mo, and S atom can also be resolved in the hexagonal packing arrangement from the Fourier filtered HRTEM images of GQD and few-layer MoS_2_, as shown in Fig. 1(i,j). These images were processed from the raw HRTEM images (indicated by red square area) in Fig. 1(e,f) by using Fourier mask filtering method [18, 19]. As shown in Fig. 1(g,h), the line profiles of contrasting intensities along the zigzag direction reveal a hexagonal lattice spacing of ~2.5 Å and ~3.2 Å for the GQD and few-layer MoS_2_, respectively. On the other hand, analysis of the contrasting intensities along the armchair direction shows the C atoms and Mo-S atoms is separated by ~1.4 Å and ~2.4 Å, respectively.

### Transmittance and electrical measurements of the fabricated devices

**Figure 2:**
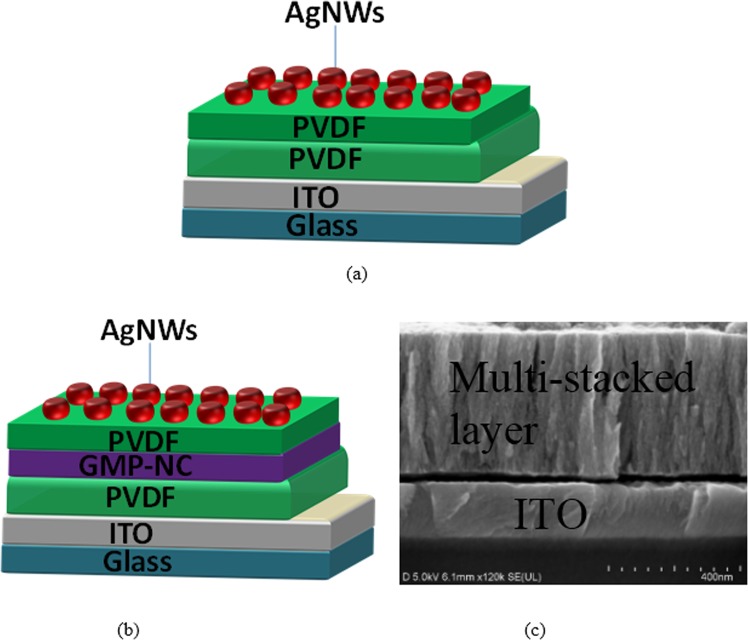
Schematic diagram of the sandwiched MIM device. (**a**) Reference device and (**b**) tristable switching device. (**c**) SEM cross-sectional structure characterization for the tristable switching device.

**Figure 3:**
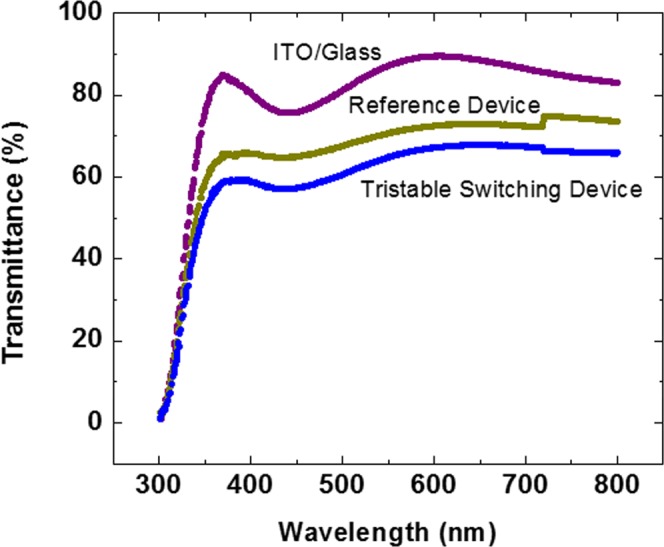
Absolute transmittance measurements of the tristable switching (blue dash line) and the reference (dark yellow dash line) devices in the visible region.

**Figure 4:**
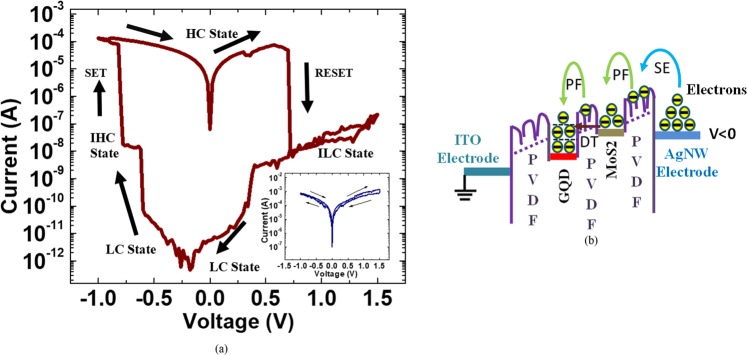
I–V hysteresis window for the fabricated tristable switching device in the scanned voltage range from −1.0 to 1.5 V and vice versa. The inset shows the negligible hysteresis window for the reference sample. (**b**) Sketch of electronic structure to illustrate the various possible dominant conduction mechanisms in the fabricated tristable switching device under the influence of an applied voltage.

Figure 2(a,b) depict the sandwiched structure of the reference and tristable switching devices. Figure 2(c) presents a SEM cross-section image of the tristable switching device with multi-stacked layer on ITO layer. Figure 3 shows the transparency measurements for the fabricated reference and tristable switching-device when compared to the ITO/glass substrate in the visible spectral range from 400 to 800 nm. The plot of purple dash line indicating the percentage of absolute transmittance for ITO/glass substrate varies from 80.4 to 89.5%, whereas the blue and dark yellow dash lines show the absolute transmittances change from 58.8 to 67.8% and 65.7 to 74.9% for the tristable switching device and reference device, respectively. The overall transparency of tristable switching device decreases approximately 20% in comparison with the ITO/glass sample. Subsequently, current-voltage (I–V) characteristics of tristable switching-devices were conducted to examine the tristable behaviour in the presence of GMP-NC layer as shown in Fig. 4(a). It is assumed that the device is in a lowest-conductivity (LC) state before the voltage sweeps as shown in Fig. 4(a). When the applied voltage is swept from 0 to −1.0 V on top of AgNWs electrode, the current increases until the first abrupt rise at −0.6 V to bring the initial LC state to an intermediate high-conductivity (IHC) state. As the voltage is further increased to −0.8 V, the device is switched to highest-conductivity (HC) state at that subsequent current rise. The observation of more than one high-conductivity state during the SET process inherents the presence of heterostructure due to the mixture of nanoparticles and flakes in the charge trapping layer^26^. The current is then maintained at HC state from −1.0 to 0 V. It is continued to be retained at HC state when the power is off, indicating nonvolatility behavior of the device. After the zero-volt, as can be seen in positive bias region in Fig. 4(a), the device can be RESET to lowest-conductivity (LC) state region by sweeping the voltage from 0 to 1.5 V. Two abrupt current reductions are observed at 0.68 V and 0.4 V, respectively. The device is primarily switched to intermediate low-conductivity (ILC) state at 0.68 V, and maintained at ILC state when the voltage increases from 0.68 to 1.5 V and return to 0.68 V. Continuing to reduce the voltage from 0.68 V, the device encounters another abrupt decrease in the current at 0.4 V and the reduced rate slows down at about 0.3 V and eventually returned to the LC state. The device demonstrates tristable switching behaviour as there are two-stage of SET and RESET processes occur before the device reached its HC and LC states. The presence of tristable switching memory window with high ON/OFF~10^7^ in Fig. 4(a) could be strongly attributed to the presence of MoS_2_-GQDs heterostructure in the tristable switching device as there is a negligible storage capability for the reference device without the integration of both MoS_2_ and GQDs as shown in the inset. The higher density of the charge storage capability for the fabricated device is believed to be enhanced by the presence of GQDs as it is not observed in the previous reported studies^17,27–30^ when only MoS_2_ flakes are introduced in the device as summarized in Table 1. The large memory hysteresis window at low-voltage further proves that the GQD enhances the charge storage capability because when the size of nanographene shrinks down to a few nm, there is a less significant band-gap induced by the quantum size effect. Consequently, its high work function and small Bohr radius makes it a favorable charge trapping material^31^. Interestingly, our study is in good agreement with the reported work by Bessonov *et al*.^26^ as shown in Table 1 when MoO_x_-MoS_2_ heterostruture was used as a charge trap layer to obtain a large ON/OFF ratio.

**Table 1:** Summary of recent progresses on MoS_2_-based non-volatile memory study.

Charge Trap Layer	Bottom/top Electrode	Turn on voltage (V)	On/off ratio	Electron transport mechanisms	Retention (s)	References
MoS_2_-PVP	ITO/Reduced grapheme oxide (RGO)	3.5	10^2^	Thermionic emission-SCLC-Ohmic	—	Liu *et al*.^11^
MoS_2_-GO	ITO/Al	<1.5	10^2^	SCLC- Ohmic-Oxygen migration	—	Yin *et al*.^21^
2H-MoS_2_-PVP	ITO/Al	<1.0	10^2^	Thermionic emission-SCLC- Ohmic	—	Zhang *et al*.^22^
MoS_2_ nanosphere	ITO/RGO	2	10^4^	Thermionic emission-SCLC- Ohmic	10^4^	Xu *et al*.^23^
MoS_2_-PMMA	ITO/Copper	2	10^4^	Ohmic-SCLC-Conducting filament	10^5^	Bhattacharjee *et al*.^24^
Heterostructure MoO_x_-MoS_2_	AgNW/Ag	0.2	>10^6^	Oxygen vacancies	10^4^	Bessonov *et al*.^20^
PVDF-MoS_2_-GQD	AgNw/ITO	0.65, 0.8	10^7^	Schottky emission-PF emission-TCLC-Ohmic	10^4^	Our present work

## Discussion

Towards the development of miniaturized electronic gadgets based on polymers, it is essential to understand the nature of several possible conduction mechanisms in dielectrics stacking structure under the action of various voltage strength regions. For ease of understanding, Fig. 4(b) shows the assumption that PVDF layer exists in between MoS_2_-GQDs heterostructure, which explains the possible occurrence of tristable switching. We believe that if the trapped charges occur solely due to the MoS_2_ or GQDs NPs, it is unlikely to generate multilevel switching but merely bistable switching states as given by the examples summarized in Table 1. Additionally, the structure of MoS_2_-GQDs without PVDF layer might be present in the nanocomposite mixture. Nevertheless, the tristable switching behavior of MoS_2_-GQDs without PVDF layer is improbable to be observed during the electrical measurement but merely a bistable switching behaviour as reported by Simone *et al*.^18^. By plotting the obtained I–V data in Fig. 4(a) on a double log scale followed by a curve fitting method, it is possible to speculate possible dominant conduction mechanisms in dielectric device based on the best fitted transportation expression. Figure 4(b) depicts the electronic structure to describe the possible dominant conduction mechanisms occurred through the fabricated tristable switching devices under the action of various strength of an applied voltage. Notice that the electrons injection is likely to be contributed from AgNWs electrode due to a lower work function relative to ITO electrode and thinner dielectric layer adjacent to it.

In Fig. 4(b), initially, at low-voltage region, it is common to observe thermally-induced electrons from AgNW electrode crossing over the PVDF barrier via thermionic emission^32^. Field enhanced thermionic emission, which is also known as Schottky emission (SE), occurs simultaneously to emit the electrons in the same voltage region when negative voltage is applied. Figure S2(a) shows the curve fitting plot of Schottky effect at T = 300 K. As the thickness of PVDF adjacent to the top electrode is measured at approximately 120 nm, the quantum tunneling is almost unlikely to occur over such a large barrier distance as the wave function of the electron is simply insufficient in real space^33^. Consequently, the emitted electrons that transported over an AgNW/PVDF interface will be trapped in the adjacent PVDF layer before they reach the first trapping site form by MoS_2_ flake. Hence, Poole-Frenkel (PF) conduction is expected to be dominant when there is a large trap density in the thick insulating thin film. The electrons can be detrapped from the PVDF layer by virtue of a lowered trap depth to reach the primary trap sites formed by MoS_2_ because of the applied voltage^4^. Figure S2(b) shows the plot of well-fitted data of PF mechanism. A sudden increase of current is observed at 0.6 V when the MoS_2_ trap sites is filled by electrons through trapped-charge limited current (TCLC) conduction mechanism because the measured slope is approximately 27.4^34^. After the first trap sites are filled with TCLC conduction, the device has achieved an IHC state. Meanwhile, it is reasonable to assume that there is a PVDF insulator layer is in between MoS_2_ and GQD. Thus, the beginning of another charge trapping in GQD trap sites could be occurred via PF emission or direct tunneling (DT) conduction. In the occurrence of PF emission, it is presumed that the thickness of the PVDF insulator in between MoS_2_ and GQD is greater than 4 nm. Alternatively, direct tunneling (DT) will take part as the dominant transportation over the PF with the electrons contribution are from MoS_2_ traps if the PVDF thickness is less than 4 nm^18,35^ before the onset of the next charge trapping in GQDs site. In Fig. 4(b), it can be seen that various electron trapping levels occurred in the GQD through TCLC conduction at −0.8 V with a measured slope of 84.4^9^. This sudden increase of the density of free carriers might be happened via PF conduction or merely DT conduction. Lastly, when the multilevel traps are fully occupied by electrons, the device enters a HC state to obey ohmic conduction as the slope is measured to be 0.98, possibly attributed to the formation of a local filament path in the device^35^.

**Figure 5:**
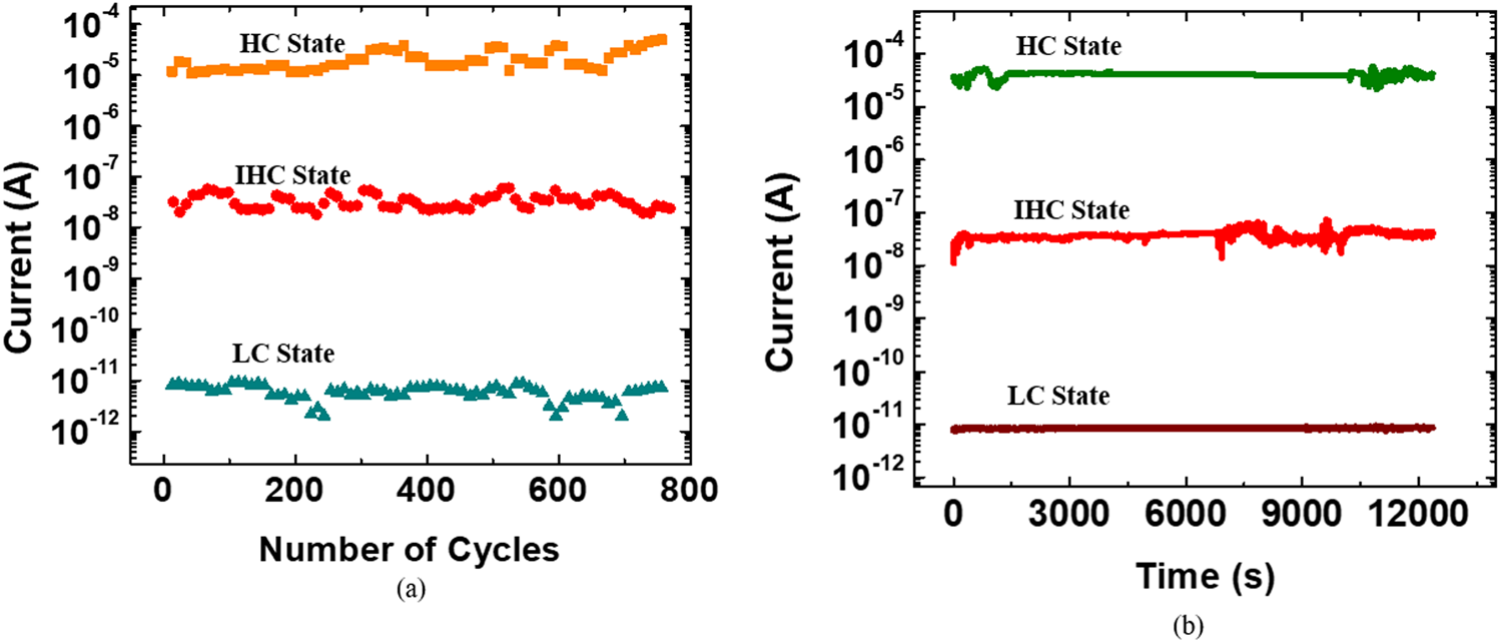
(**a**) Endurance cycle and (**b**) retention tests were conducted to examine the stability performance of the fabricated tristable switching device.

The ruptures of local filament at about 0.68 V and 0.4 V could be the reason for the two-stage Reset process observation^4^. It is worth to mention that there is a slight increase in current from 0.68 to 1.5 V because a higher strength of voltage is required to remove the electrons from the various electron trapping levels in the GQD. The subsequent reduction in current during the reverse swept of voltage from 1.5 to 0 V is probably owed to breakage of conductive paths. This is the reason that we speculate the charges are first trapped in MoS_2_ trap sites and then in GQDs various electrons levels trap sites during the two-stage Set process as aforementioned. An endurance cycle was carried out to demonstrate the repeatability of the device for 750 SET–RESET cycles as shown in Fig. 5(a). The sequence of “SET I (−0.7 V) – Read (−0.25 V) – SET II (−1.0 V) – Read (−0.25 V) – RESET (1.5 V) – Read (−0.25 V)” was applied to the device. IHC, HC, and LC states can be achieved by applying SET I, SET II and RESET voltages to the fabricated device. Figure 5(b) shows that retention test was also conducted to the fabricated device using the identical Set and Reset voltages under ambient conditions for 1 × 10^4^ s. It is found that the three-conductivity states of the tristable switching device are stable and distinguishable over the time when read at −0.25 V.

This work demonstrates a high-density storage of tristable switching device using simple solution fabrication route. The multilevel switching behavior is deduced to the presence MoS_2_-GQDs heterostructure in the charge trapping layer of device. TEM characterizations of atomic structure for GQDs-MoS_2_ heterostructure suggest that the MoS_2_ flakes can be simply nano-engineered with the GQDs by our dispersion method. 10^7^ ON/OFF ratio was achieved for the obtained hysteresis window. I–V curve fitting was performed to identify the possible dominant electrical transportations through the device by the obtained data. The three conductivity states for the device are distinguishable and remained stable for 1 × 10^4^ s when endurance cycle and retention tests were performed, respectively. Thus, a very simple three-layer stacked structure with GQDs-MoS_2_ heterostructure as a high charge trapping layer could be a right step to fulfill the current market trend towards miniaturized high information storage electronic products.

## Methods

### Preparation of GMP-NC mixture

1 mg/mL of GQDs in deionized water and MoS_2_ flakes in ethanol/water were purchased from ACS Material and Graphene Supermarket, respectively. A 250 mg of PVDF powder purchased from Sigma Aldrich was dissolved in a 10 mL dimethylformamide at 75 °C. Then we stirred the solution for 2 h to prepare the PVDF solution^36^. Meanwhile, the GMP-NC mixture was prepared by mixing each 1 mL of GQDs and MoS_2_ NPs solutions respectively into the 0.5 mL of PVDF solution and followed by 15 min ultrasonication of the mixture to ensure homogeneous distribution of the GQDs and MoS_2_ NPs in the PVDF matrix.

### Fabrication of tristable switching devices

The reference and tristable switching devices were fabricated in the structure of (a) silver nanowires (AgNWs)/PVDF/PVDF/ITO/glass and (b) AgNWs/PVDF/GMP-NC/ PVDF/ITO/glass, respectively as shown in Fig. 2(a,b). Prior to device fabrication, the 1.5 cm × 1.0 cm ITO/glass substrates were ultrasonically cleaned according to the cleaning procedures described by Ooi *et al*.^4^. First, a 160 nm thick PVDF layer was deposited on top of ITO/glass substrate at 2000 rpm for 40 s and heated at 140 °C for 1 h. Then, a 140 nm GMP-NC layer was deposited at 2000 rpm for 40 s and heated under the same conditions of temperature and duration. Subsequently, a 120 nm PVDF layer was deposited using parameters of 3000 rpm for 40 s and heated at 140 °C for 1 h. Lastly, the AgNWs top electrodes were deposited following the reported method in literatures^4,9^.

### Characterization

All electrical measurements were measured by a semiconductor characterization system (Keithley 4200-SCS). The bias voltage was applied on top of AgNW top electrode and the ITO bottom electrode was grounded. An ultraviolet–visible spectroscopy system (Agilent Cary 7000) was used to characterize the transmittance of the tristable switching devices. A field emission scanning electron microscopy (FESEM) system (JEOL, JSM 7500 F) was conducted to characterize the cross-section images of the constructed devices. The lattice-resolved images of GQDs-MoS_2_ heterostructure were obtained by high-resolution transmission electron microscopy (HRTEM) system (JEOL, JEM-2010) operated at an accelerating voltage of 200 kV.

## Supplementary information

Supplementary Information for Electrical Transportation Mechanisms of Molybdenum Disulfide Flakes-Graphene Quantum Dots Heterostructure Embedded in Polyvinylidene Fluoride Polymer
