# Supplementary material for: Electrical transportation mechanisms of molybdenum disulfide flakes-graphene quantum dots heterostructure embedded in polyvinylidene fluoride polymer
Source: Sci Rep. 2019 May 1;9:6761. doi: 10.1038/s41598-019-43279-3 (PMC6494838; doi:10.1038/s41598-019-43279-3)
Supplement: Supplementary file 1 — Supplementary Information for Electrical Transportation Mechanisms of Molybdenum Disulfide Flakes-Graphene Quantum Dots Heterostructure Embedded in Polyvinylidene Fluoride Polymer [file 41598_2019_43279_MOESM1_ESM.docx]

Supplementary Information for Electrical Transportation Mechanisms of Molybdenum Disulfide Flakes-Graphene Quantum Dots Heterostructure Embedded in Polyvinylidene Fluoride Polymer

Poh Choon Ooi^1,*^, Muhammad Aniq Shazni Mohammad Haniff ^2^, M. F. Mohd. Razip Wee ^1,*^
 Boon Tong Goh^3^, Chang Fu Dee^1,*^, Mohd Ambri Mohamed^1^, Burhanuddin Yeop Majlis^1^

^1^Institute of Microengineering and Nanoelectronic, Universiti Kebangsaan Malaysia,
43600 Bangi, Malaysia

^2^Advanced Devices Lab, MIMOS Berhad, Technology Park Malaysia,
57000 Kuala Lumpur, Malaysia

^3^Low Dimensional Materials Research Centre (LDMRC), Department of Physics,
Faculty of Science, University of Malaya, 50603 Kuala Lumpur, Malaysia

^*^Correspondence and requests for materials should be addressed to P.C.O. (pcooi@gmx.com); C.F.D. (cfdee@ukm.edu.my); M.F.M.R. (m.farhanulhakim@ukm.edu.my)


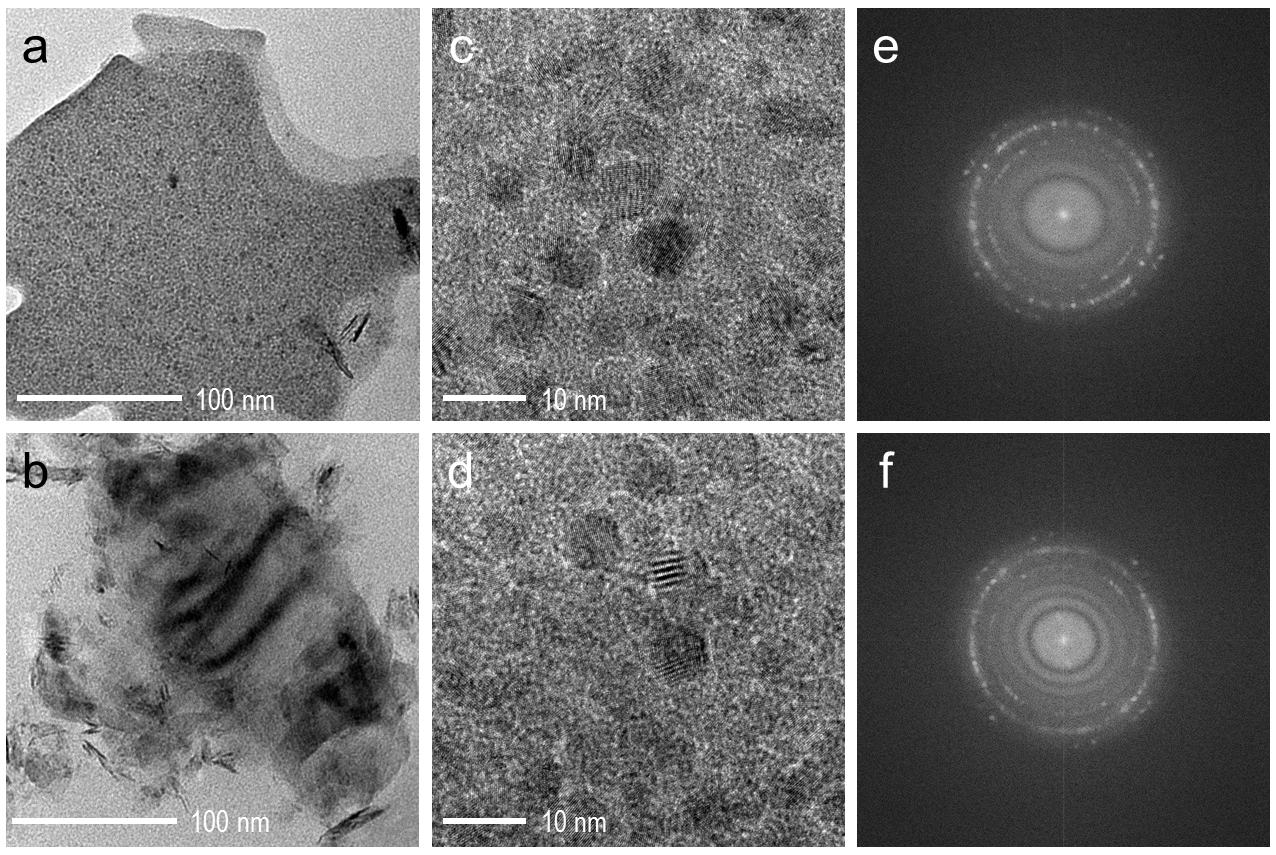


**Figure S1** Characterization of GQDs-MoS_2_ heterostructure. (a-b) TEM images of GQDs-MoS_2_ heterostructure. (c-d) HR-TEM images of GQDs on the MoS_2_ flakes in random orientation showing uniformly-distributed sizes and shapes. (e-f) The corresponding FFT pattern of GQDs-MoS_2_ heterostructure showing typical Debye-Scherrer type ring pattern.

(b)

(a)

**Figure S2** Electrical conductivity fitting of (a) Schottky emission and (b) PF emission in low voltage region using the obtained I-V data as plotted in Figure 4 by setting T=300 K.
